# Effective Long Afterglow Amplification Induced by Surface Coordination Interaction

**DOI:** 10.1002/advs.202306942

**Published:** 2023-12-31

**Authors:** Yongkang Wang, Qiankun Li, Lunjun Qu, Jiayue Huang, Ying Zhu, Chen Li, Qingao Chen, Yan Zheng, Chaolong Yang

**Affiliations:** ^1^ School of Materials Science and Engineering Chongqing University of Technology Chongqing 400054 China; ^2^ Guangdong Provincial Key Laboratory of Luminescence from Molecular Aggregates South China University of Technology Guangzhou 510640 China

**Keywords:** afterglow amplification, long‐persistent luminescence, low‐vision lighting, organic–inorganic hybrid, surface coordination, triplet energy transfer

## Abstract

Long‐persistent luminescent (LPL) materials have attracted considerable research interest due to their extensive applications and outstanding afterglow performance. However, the performance of red LPL materials lags behind that of green and blue materials. Therefore, it is crucial to explore novel red LPL materials. This study introduces a straightforward and viable strategy for organic–inorganic hybrids, wherein the organic ligand 1,3,6,8‐Tetrakis(4‐carboxyphenyl)pyrene (TCPP) is coordinated to the surface of a red persistent phosphor Sr_0.75_Ca_0.25_S:Eu^2+^ (R) through a one‐step method. TCPP serves as an antenna, facilitating the transfer of absorbed light energy to R via triplet energy transfer (TET). Notably, the initial afterglow intensity and luminance of R increase by twofold and onefold, respectively, and the afterglow duration extends from 9 to 17 min. Furthermore, this study involves the preparation of a highly flexible film by mixing R@TCPP with high‐density polyethylene (HDPE) to create a sound‐controlled afterglow lamp. This innovative approach holds promising application prospects in flexible large‐area luminescence, flexible wearables, and low‐vision lighting.

## Graphical abstract

Outstanding red long‐persistent luminescence materials are achieved through surface coordination interactions between organic ligands and inorganic phosphors. Remarkably, the afterglow performance of this material experienced significant enhancement due to the interplay of triplet energy transfer and the trap effect.

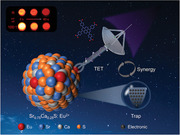

## 1 Introduction

Long‐persistent luminescent (LPL) materials exhibit the ability to store light energy and release it through long‐lasting afterglow emission.^[^ ^1^, ^2^ ^]^ Broadly, LPL materials can be categorized into inorganic long‐persistent luminescent (ILPL) and organic long‐persistent luminescent (OLPL) materials. Among inorganic systems, the green ILPL phosphor SrAl_2_O_4_:Eu^2+^, Dy^3+^ (SAOED), with an impressive afterglow duration of up to 30 h was initially reported in 1996.^[^ ^3^ ^]^ Since then, this breakthrough has catalyzed the rapid advancement of ILPL materials. Numerous ILPL materials have subsequently been developed and found widespread use in applications such as anti‐counterfeiting,^[^ ^4^, ^5^ ^]^ optical information storage,^[^ ^6^, ^7^ ^]^ photocatalysis,^[^ ^8^ ^]^ and sensing.^[^ ^9^, ^10^ ^]^ Organic systems achieve LPL by promoting intersystem crossing (ISC) and suppressing non‐radiative transitions through mechanisms like the heavy‐atom effect,^[^ ^11^ ^]^ carbonyl functional groups,^[^ ^12^ ^]^ introduction of a rigid polymer matrix,^[^ ^13^ ^]^ and cross‐linking strategies.^[^ ^14^ ^]^ OLPL materials, known for their ease of synthesis and the ability to modify targeted functionalities and processes, are extensively applied in diverse fields such as biological imaging,^[^ ^15^ ^]^ dynamic anti‐counterfeiting,^[^ ^16^ ^]^ and organic light‐emitting diodes.^[^ ^17^ ^]^ However, the development of LPL materials faces challenges, including the absence of luminescent centers and matrices, resulting in red ILPL materials exhibiting inferior afterglow performance compared to their green and blue counterparts. Therefore, the exploration and enhancement of red LPL materials hold paramount significance.

To date, improvements in the performance of red inorganic persistent phosphors have primarily centered around discovering new, suitable matrices, and co‐doping with metal ions.^[^ ^18^, ^19^, ^20^, ^21^, ^22^, ^23^, ^24^ ^]^ Regrettably, these endeavors have not yielded significant enhancements in their performances. Organic–inorganic hybrid strategies have garnered substantial interest among researchers due to their potential to effectively integrate the merits of both organic and inorganic materials. However, the existing body of research on organic‐inorganic hybridization predominantly focuses on configurations such as organic ligand@carbon dots^[^ ^25^, ^26^, ^27^ ^]^ and organic ligand@persistent luminescence nanocrystals,^[^ ^28^ ^]^ most of which exhibit up‐conversion luminescence. Notably, hybridizing organic ligands with persistent inorganic phosphors has been scarcely reported. Furthermore, persistent inorganic phosphors are hindered by a disadvantageous lack of light‐absorption ability. In contrast, organic ligands, especially pyrene and its derivatives, demonstrate outstanding light‐absorption capabilities compared to their inorganic persistent phosphor counterparts. Therefore, a viable strategy for modifying and enhancing the afterglow performance of red inorganic persistent phosphors lies in combining the advantageous features of both organic ligands and inorganic persistent phosphors.

**Figure 1:**
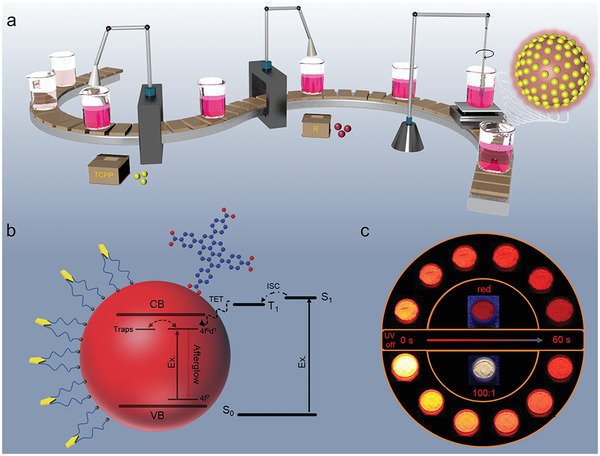
Schematic diagram of OILPL material. a) Schematic diagram of the preparation process of OILPL materials. b) Schematic diagram of energy transfer mechanism and coordination of OILPL materials. c) Afterglow photos of R and R@TCPP 100:1 within 60 s after excitation with 365 nm UV lamp (UV lamp power is 5 W, irradiation time is 5 s).

In this investigation, an organic‐inorganic long‐persistent luminescent (OILPL) material was conceived and crafted by merging a commercially established red inorganic persistent phosphor, Sr_0.75_Ca_0.25_S: Eu^2+^ (R), with the organic ligand 1,3,6,8‐tetra(4‐carboxyphenyl)pyrene (TCPP). TCPP, characterized as a macrocyclic conjugated carboxylic acid with remarkable light‐absorbing capabilities, can establish effective surface coordination interactions with the phosphor R. Consequently, it serves as an antenna, absorbing light radiation and transferring energy to R through triplet energy transfer (TET) (**Figure** **1**a,b). Notably, the initial afterglow intensity and luminance of the adorned R@TCPP 100:1 surpassed those of the pure red inorganic persistent phosphors by two and one time, respectively. Moreover, the afterglow duration was nearly one time longer than that of the unperturbed red inorganic persistent phosphors (Figure 1c; Movie S1, Supporting Information), showcasing an enhancement in performance.

## 2 Results and Discussion

**Figure 2:**
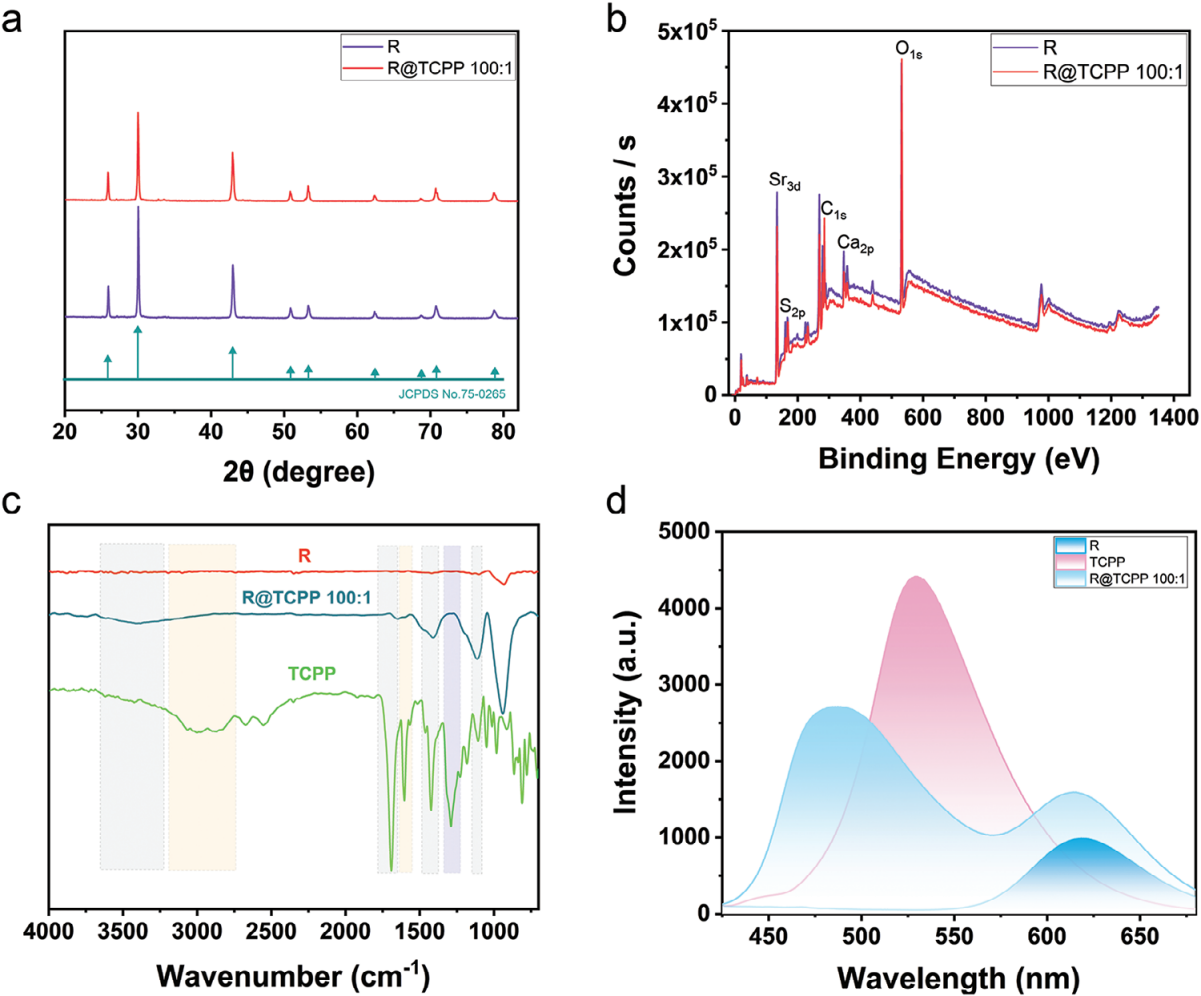
Structure, elemental characterization of OILPL material. a) XRD spectra of R, R@TCPP 100:1 and standard card (JCPDS NO. 75–0265). b) XPS spectra of R and R@TCPP 100:1. c) FT‐IR spectra of R, TCPP, and R@TCPP 100:1. d) Fluorescence spectra of R, R@TCPP 100:1 and TCPP (λex = 365 nm).

The preparation method for this material is remarkably convenient and straightforward. It involves the direct addition of phosphor R and the ligand TCPP into beakers at various mass ratios, followed by heating and mixing (Figure 1a). To validate the structure and composition of the prepared materials and confirm the successful incorporation of R and TCPP, X‐Ray Diffraction (XRD), X‐Ray Photoelectron Spectroscopy (XPS), and Fourier‐transform infrared spectroscopy (FT‐IR) analyses were conducted on R, TCPP, and R@TCPP 100:1. The XRD spectra revealed that the crystal structures of R and R@TCPP 100:1 remained consistent, with all diffraction peaks aligning with standard data (JCPDS No.75‐0265)^[^ ^29^ ^]^ (**Figure** **2**a). This indicated that the inclusion of R and the ligand TCCP did not alter the crystal structure of R. XPS analysis of the near‐surface chemical compositions of R and R@TCPP 100:1 identified elements C, O, S, Ca, and Sr, along with trace amounts of Eu (Figure 2b; Figures S1 and S2, Supporting Information). The C and O elements in R may have originated from unsulfurized CaCO_3_ during its preparation, as evidenced by fitting peaks of C_1s_ from CaCO_3_ found in the Ca fitting spectra of R and R@TCPP (289.38 and 289.41 eV). Notably, the C_1s_ and O_1s_ peak values, as well as the percentage contents of C and O in R@TCPP 100:1, were significantly higher than those in R (Figure 2b; Table S1, Supporting Information), attributed in part to the C and O in TCPP. In the FT‐IR spectra of R and R@TCPP 100:1 (Figure 2c), characteristic peaks from the carboxyl group (3400, 1691, 1400, and 1100 cm^−1^) and the benzene ring (3000 and 1604 cm^−1^) were evident in the R@TCPP 100:1 spectrum. With an increasing proportion of organic ligands in the R@TCPP series of samples, the characteristic peaks of the ligand TCCP became more pronounced (Figure S3, Supporting Information).

Additionally, fluorescence spectroscopy was conducted, revealing a single emission peak from R at 625 nm, corresponding to the 4f^6^d^1^‐4f^7^ transition of Eu^2+^. Unexpectedly, the spectra of the R@TCPP series samples exhibited a novel fluorescence emission peak at 470 nm. The emission intensity gradually increased with the augmentation of ligand TCPP, and the fluorescence color shifted from orange‐red to a vibrant cyan (Figure 2d; Figure S4, Supporting Information). TCPP's fluorescence peak was observed at 530 nm, representing a 60 nm shift from the aforementioned emission peak. This shift indicated the successful coordination of TCPP to the surface of phosphor R through the abundant carboxyl groups. The coordination altered the energy level of TCPP, leading to a noticeable blue shift in the fluorescence emission peak of R@TCPP.

For visual confirmation of coordination, scanning electron microscopy (SEM) images of R and R@TCPP 100:1 were acquired (Figure S5, Supporting Information). The outcomes indicated a notable particle aggregation in R@TCPP 100:1 compared to R. This aggregation was attributed to the presence of free carboxyl groups on the surface of R@TCPP 100:1, with interactions between these carboxyl groups causing the particles to attract each other. Consequently, the SEM images provided additional confirmation of the successful surface coordination of R and TCPP.

**Figure 3:**
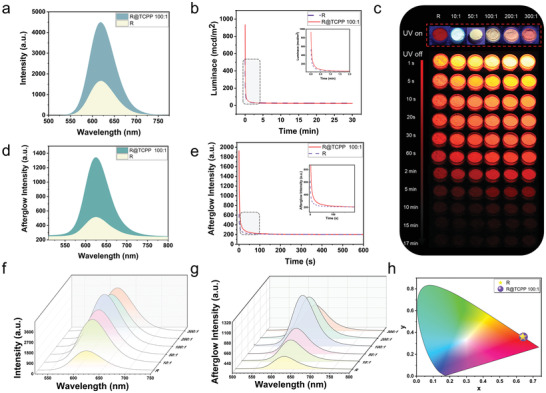
a) Photophysical properties of OILPL materials. a. Phosphorescence spectrum of R and R@TCPP 100:1 (λ_ex._ = 365 nm). b) Afterglow luminance decay curves of R and R@TCPP 100:1 (λ_ex._ = 365 nm), with a partially enlarged detail image in the top right. c) Afterglow imaging of R and R@TCPP at various ratios (λ_ex._ = 365 nm, UV lamp power: 5 W, irradiation time: 5 s). d) Afterglow spectrum of R and R@TCPP 100:1. Afterglow spectra were obtained by scanning the sample five times within 10 s after removing the UV lamp. The figure displays the spectrum from the first scan (λ_ex._ = 365 nm, UV lamp power: 30 W, irradiation time: 5 s). e) Afterglow intensity decay curves of R and R@TCPP 100:1 (λ_ex._ = 365 nm, UV lamp power: 30 W, irradiation time: 30 s), with a partially enlarged detail image in the top right. f) Phosphorescent spectra of R and R@TCPP at various ratios (λ_ex._ = 365 nm). g) Afterglow spectrum of R and R@TCPP at various ratios. The figure shows the spectrum from the first scan (λ_ex._ = 365 nm, UV lamp power: 30 W, irradiation time: 5 s). h) CIE coordinate diagram corresponding to (a).

The photophysical properties of R and R@TCPP were meticulously investigated (**Figure** **3**; Figures S6–S8, Supporting Information). Due to the superior enhancement observed in R@TCPP 100:1, this particular ratio was chosen as an illustrative example for a more in‐depth discussion. Analysis of the phosphorescence and afterglow spectra revealed a nearly threefold increase in the phosphorescence intensity of R@TCPP 100:1 compared to R (from 1661.0 to 4497.0 a.u.). Moreover, the intensity of the afterglow spectra exhibited a more than twofold increment, rising from 625.0 to 1344.0 a.u. (Figure 3a,d). Both R and R@TCPP 100:1 exhibited emission peaks in the afterglow and phosphorescence spectra at 625 nm, and CIE chromaticity diagrams confirmed substantial overlap in their color coordinates (x = 0.64, y = 0.35 and x = 0.63, y = 0.36, respectively) (Figure 3h; Figure S9, Supporting Information). The phosphorescence and afterglow, stemming from the radiative transition of Eu^2+^, demonstrated that the ligand TCPP lacked the ability to emit phosphorescence at room temperature. However, it exhibited phosphorescence emission at 525 and 700 nm at 77 K (Figure S10, Supporting Information). Examination of the afterglow luminance and intensity decay spectra of R and R@TCPP 100:1 revealed a significant increase in the initial luminance of the sample from 531.5 to 934.0 mcd m^−2^, along with a rise in the initial intensity from 617.1 to 1927.0 a.u. (Figure 3b,e). Consequently, the incorporation of TCPP on the surface of R markedly enhanced its light absorption ability.

To examine the impact of the R to TCPP ratio on afterglow performance, a series of samples with different ratios were prepared, labeled as R@TCPP 10:1, R@TCPP 50:1, R@TCPP 100:1, R@TCPP 200:1, and R@TCPP 300:1. As the proportion of TCPP increased, both phosphorescence intensity (3680.0, 3855.0, 4497.0, 3808.0, and 3715.0 a.u.) and afterglow intensity (705.0, 795.0, 1344.0, 1140.0, and 802.0 a.u.) initially rose and then declined. Intriguingly, the emission intensity of all decorated samples surpassed that of R, with R@TCPP 100:1 exhibiting the most robust afterglow performance (Figure 3c,f,g). The effects of different proportions on afterglow performance can be attributed to two factors. First, an excessive addition of TCPP can detrimentally impact the light absorption ability of R, thereby restricting afterglow emission. The optimal mass ratio was determined to be 100:1 (w/w). Second, interaction forces exist between TCPP molecules due to the presence of a large ring and the multi‐carboxylic structure of TCPP. The higher the proportion of ligand TCPP, the more pronounced the clustering effect in the samples (Figure S11, Supporting Information). In conclusion, the coordination interaction between TCPP and R effectively facilitates energy transfer, thereby enhancing the afterglow performance of the system.

**Figure 4:**
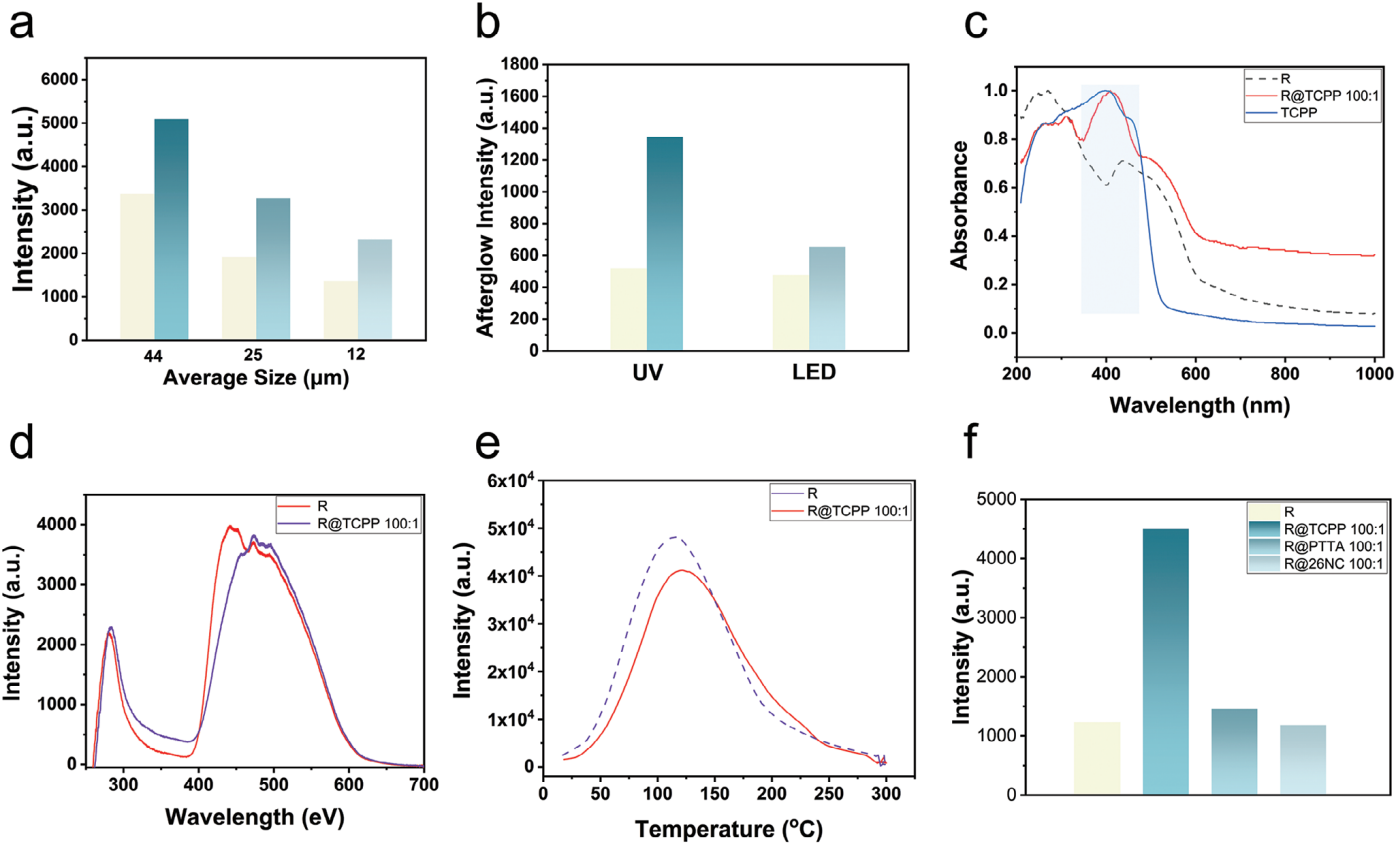
Study on factors affecting amplification of organic‐inorganic long persistent luminescence materials. a) Comparison of phosphorescence intensity before and after R modification with different average particle sizes (λ_ex._ = 365 nm), the mass ratio of R and TCPP was 100:1 for all three average particle size cases. b) Comparison of the afterglow intensity of R and R@TCPP 100:1 after excitation by UV and LED lamps (The wavelength of the UV lamp is 365 nm, the power is 30 W, LED from smartphones, irradiation time is 5s, the figure shows the spectrum obtained from the first scanning). c) Normalized UV–vis absorption spectra of R, R@TCPP 100:1 and TCPP. d) Excitation spectrum of R and R@TCPP 100:1(λ_em._ = 625 nm). e) Thermoluminescence spectrum of R and R@TCPP 100:1 (Heating rate is 1 °C s^−1^). f) Comparison of phosphorescence intensity of R, R@TCPP 100:1, R@PTTA 100:1 and R@26NC100:1.

The coordination between phosphor R and TCPP is influenced by both the ratio of R to TCPP and the particle size of R. To investigate the impact of particle size on coordination interaction, phosphor R was ground and divided into three average particle sizes: 44, 25, and 12 µm. Subsequently, TCPP was introduced for modification. Following the coordination of R and TCPP at different particle sizes (mass ratio of 100:1), the phosphorescence intensities of the samples were enhanced by 51.5%, 71.5%, and 70.7%, respectively (**Figure** **4**a; Figures S12–S14, Supporting Information). Notably, smaller particle sizes led to lower phosphorescence intensities for both R and the modified R. The enhancement effect of R was low even after modification. Because the high‐temperature solid‐phase method of the synthesized R had a high firing temperature, long reaction time, large grain size, high density, and high product hardness, the larger the particle size of the product, the higher the brightness. After grinding, the integrity of the crystals of the R was affected, which led to a decrease in its phosphorescence intensity. Interestingly, despite the reduction in particle size, the enhancement effect improved. This phenomenon can be attributed to two factors. Firstly, as the particle size decreased, the specific surface area of the sample increased, exposing more coordination sites of Eu^2+^ on the surface of R. This increased the number and success rate of coordination sites, facilitating energy transfer. Secondly, smaller particle sizes promoted a more uniform distribution of TCPP on the surface of R, reducing the interaction force between ligands and enhancing energy transfer efficiency. However, excessively small particle sizes could dominate negative effects on luminescence. For practical applications, inorganic persistent phosphors are ideally kept at a small particle size. The strategy employed in this study holds promise for significantly improving the afterglow performance of R with small particle size, providing a potential solution to this issue.

To assess the sensitivity of R@TCPP to UV light post‐incorporation, afterglow spectra of R and R@TCPP 100:1 were acquired using various excitation sources (Figure 4b; Figures S15 and S16, Supporting Information). Under 365 nm UV lamp excitation, the afterglow intensity of R@TCPP 100:1 increased by nearly twofold compared to that of R, while the increase was only 37.5% when LED lamps were employed as the excitation source. These findings underscore that the coordination interaction with TCPP renders phosphor R more responsive to UV light. The absorption of UV light was significantly enhanced post‐incorporation with TCPP, effectively improving the afterglow performance of R. In the UV–vis absorption spectra of TCPP, R, and R@TCPP 100:1, R@TCPP 100:1 displayed a new absorption peak at 330–450 nm (Figure 4c), attributable to the π–π* transition of C═C on the highly conjugated aromatic ring, a characteristic of TCPP. Notably, the excitation spectrum of R and R@TCPP 100:1 (λ_em._ = 625 nm) revealed a significantly higher peak intensity in the 300–400 nm range for R@TCPP 100:1 compared to R, aligning with the changes in the UV–vis absorption spectra of both (Figure 4d). Collectively, these results indicate that the amplification effect of the TCPP ligand contributes to the enhanced afterglow performance. Specifically, TCPP augments the absorption ability of phosphor R in the UV and near‐UV regions, transferring energy to R and substantially improving its afterglow performance.

For inorganic persistent phosphors, trap depth is a crucial factor affecting afterglow performance, with thermoluminescence spectroscopy being the predominant test method for trap depth assessment. Typically, shallow trap energy levels correspond to pyroelectric peaks at the low‐temperature end. After capturing carriers, these carriers quickly escape from the traps upon receiving thermal perturbation at room temperature, leading to an initial afterglow luminance. In contrast, electrons in deep energy levels face challenges returning to the conduction band through thermal perturbation at low temperatures. However, once thermally excited, their likelihood of escaping the trap increases, enhancing luminescence and forming thermoluminescence. The temperature corresponding to the peak of the thermoluminescence spectrum indicates the trap's depth, with higher temperatures suggesting deeper traps, smaller probabilities of electron release, longer recombination luminescence times, and extended macroscopic afterglow durations. Analysis of the thermoluminescence spectra of R and R@TCPP 100:1 reveals a single broad peak at 116 °C for R and a single broad peak at 122 °C for R@TCPP 100:1 (Figure 4e). According to Equation,^[^ ^30^ ^]^

(1) $$E=T_{m}/500$$

where *E* is the trap depth and *T*_m_ is the Kelvin temperature corresponding to the peak.

The trap depths of R and R@TCPP 100:1 were calculated to be 0.77 and 0.79 eV, respectively. Nevertheless, the XRD spectra of both samples indicated no changes in their crystal structures. Therefore, the alteration in trap depth is attributed to the following factors. The ligand TCPP absorbs light energy, transfers it to Eu^2+^ through triplet energy transfer (TET), and rapidly releases energy through radiative transitions. This process is faster than the escape of carriers from the trap, resulting in the early luminescence of R@TCPP 100:1 being predominantly derived from TCPP, not entirely from the recombination of carriers escaping the trap. The trap effect becomes dominant only after the depletion of energy from TCPP. Hence, R@TCPP 100:1 required a slightly higher temperature than R to achieve the highest thermoluminescence intensity. This also elucidates why the initial afterglow of R@TCPP 100:1 was brighter and had a longer duration. The incorporation of TCPP did not deepen the trap by altering the crystal structure of R but provided a richer energy source. The entire afterglow process resulted from the synergy between TET and the trap effect, with R@TCPP 100:1 obtaining more energy and exhibiting superior afterglow performance.

To validate the universality of the strategy, we incorporated R with 5,5′,5′′,5′′′‐(Pyrene‐1,3,6,8tetrayl) tetraisophthalic acid (PTTA) and 2,6‐Naphthalenedicarboxylic acid (26NC) and examined their photophysical properties (Figure 4f; Figures S17–S21 and Movie S2, Supporting Information). The fluorescence peaks of PTTA and 26NC exhibited a noticeable blue shift after coordination with R, indicating successful coordination. Surprisingly, the amplification effects of the three organic ligands were significantly different. The phosphorescent intensity of R@TCCP 100:1 enhanced by 266.0% compared to R, R@PTTA 100:1 enhanced only by 15.1%, while the phosphorescent intensity of R@26NC 100:1 instead weakened by 3.5%. This unexpected result prompted further investigation into the underlying luminescence mechanism of this synergistic enhancement; therefore, we conducted theoretical calculations for three small molecules (TCPP, PTTA, and 26NC), including spin–orbit coupling (SOC) and energy mapping.^[^ ^31^, ^32^, ^33^, ^34^ ^]^

**Figure 5:**
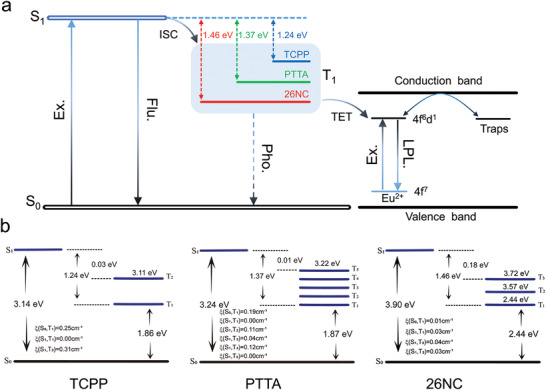
Theoretical calculations of R, R@TCPP 100:1, R@PTTA 100:1 and R@26NC. a, Mechanism diagram of R@TCPP 100:1, R@PTTA 100:1 and R@26NC. (Ex. = Excitation, Flu. = Fluorescence, Pho. = Phosphorescence, LPL = Long‐persistent luminescent). b, Energy level diagram and SOC for TCPP, PTTA, 26NC.

The calculations were conducted using time‐dependent density functional theory (TDDFT) and dispersion‐corrected density functional theory (DFT‐D3) for optimization (**Figure** **5**a,b). Regarding the energy‐exchange process at the interface between organic ligands, carbon quantum dots, and inorganic persistent luminescence nanocrystals, existing studies have generally proposed that energy must be transferred in the form of long‐lived triplet states.^[^ ^35^, ^36^, ^37^ ^]^ Consequently, the ability of organic ligands to generate triplet excitons plays a crucial role in determining the energy transfer efficiency. The S_1_ vertical excitation energies of the three organic ligands were 3.14, 3.17, and 3.90 eV, respectively. The S_1_‐T_1_ energy gaps were 1.24, 1.37, and 1.46 eV, respectively. Additionally, the spin–orbit coupling (SOC) constants of TCPP were significantly higher than those of PTTA and 26NC. Collectively, under UV excitation, photogenerated singlet excitons can be converted to triplet excitons by intersystem crossing (ISC) with unity efficiency on a sub‐10‐ps time scale for TCPP, PTTA, and 26NC. TCPP exhibited higher efficiency in generating triplet excitons due to its lower S_1_ vertical excitation energy, lower S_1_‐T_1_ energy gap, and larger SOC constant. Interestingly, the TCPP T_1_‐S_0_ energy gap (1.86 eV) was smaller than those of PTTA (1.87 eV) and 26NC (2.44 eV). An essential condition for triplet energy transfer (TET) is that the triplet energy level of organic ligands must be higher than the emission orbital energy level of Eu^2+^(4f^6^d^1^), and TCPP has the lowest triplet energy level. Speculating from the afterglow phenomena of the three ions, it is inferred that the emission orbital energy level of Eu^2+^ is slightly lower than the T_1_ energy level of TCPP. The minimal energy level difference between the T_1_ energy level of TCPP and the Eu^2+^ emitting orbital energy level not only efficiently generates triplet states but also facilitates TET, resulting in a significant afterglow enhancement of R. Importantly, these theoretical findings align with experimental results. The efficiency of triplet exciton generation in 26NC was not sufficiently high, and the energy difference between the T_1_ energy level and the Eu^2+^‐emitting orbital energy level may impede the TET process. Consequently, the absorbed energy by 26NC cannot effectively transfer to R; the coordination interaction of 26NC and R influences light absorption ability and afterglow performance, leading to the phosphorescence intensity of R@26NC 100:1 being even lower than that of R. In summary, the afterglow process of R@TCPP 100:1 was synergized by TET and trap effects. The TET process was influenced by triplet exciton generation efficiency of organic ligands and the energy gaps of T_1_ and Eu^2+^ emission orbital energy levels. A suitable energy gap and higher triplet efficiency would significantly improve TET and enhance the afterglow performance of these systems.

**Figure 6:**
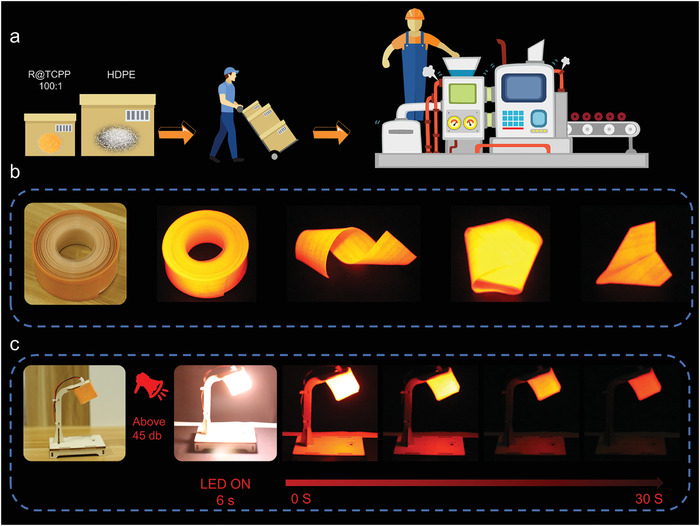
Application of OILPL materials. a) HDPE@R@TCPP 100:1 film processing flow diagram. b) Real picture and Afterglow photo of HDPE@R@TCPP 100:1 film, the afterglow photo of HDPE@R@TCPP 100:1 film that is folded into various shapes (The excitation wavelength is 365 nm, the power is 30 W UV lamp, irradiation time is 5 s), the thickness of the film is 0.29 mm and the width is 30 mm. c) the real photo of the afterglow sound control lamp, the schematic diagram of the lighting process.

Given the outstanding afterglow performance of R@TCPP 100:1, we envision its potential application in flexible large‐area luminescence. Initially, R@TCPP 100:1 and high‐density polyethylene (HDPE) were meticulously blended in a beaker at a mass ratio of 1:50 and introduced into a torque rheometer (model RM‐200A). The mixture underwent processing to yield an HDPE@R@TCPP 100:1 film, subsequently compressed into rolls (**Figure** **6**a). As anticipated, the orange‐red film emitted a red afterglow under both daylight and UV lamp excitation. Remarkably, the afterglow performance of the film remained stable even after folding into various shapes (Figure 6b), indicating that the HDPE@R@TCPP 100:1 film possesses not only excellent flexibility but also remarkable afterglow performance, sustaining an afterglow duration of up to 5 min following UV lamp excitation (Movie S3, Supporting Information)

In addition, the film's toughness properties were investigated (Figure S22, Supporting Information). The film demonstrated a maximum tensile force of 29.7 N and a tensile strength of 25.6 MPa. Interestingly, a 2.6 kg reaction kettle was lifted by the film without fracture, and the afterglow properties remained unchanged (Figures S23 and S24, Supporting Information). Expanding on this, a simple and portable sound‐controlled afterglow lamp was fabricated (Figure 6c). The HDPE@R@TCPP 100:1 film was fashioned into a square lamp with an LED serving as the excitation light source. Once the circuit was connected, if the sound‐controlled afterglow lamp detected a sound signal greater than 45 dB, the sound detection and amplification circuit amplified it. The control circuit then activated the LED light to glow. Under the control of the delay circuit, the LED light persisted for a set duration of 6 s. After the LED lamp was extinguished, the lampshade continued to emit red afterglow. The afterglow could last for 600 s, and within 40 s, the afterglow luminance of the lampshade still provided a considerable degree of weak illumination (Figure 6c; Movie S4, Supporting Information). This capability facilitates substantial electricity savings. Therefore, the light‐emitting film prepared in this study holds promising application prospects for large‐area flexible luminescence, flexible wearables, and daily low‐vision lighting.

## 3 Conclusion

In this study, we introduced an organic‐inorganic hybrid strategy to enhance the afterglow performance of red inorganic persistent phosphors. The strategy involves coordinating 1,3,6,8‐Tetrakis(4‐carboxyphenyl)pyrene (TCPP) with the red persistent phosphor R. TCPP acts as an antenna, absorbing light energy and transferring it to R. The triplet energy transfer (TET) and trap effects synergize to significantly improve the afterglow performance of R. Consequently, the initial afterglow intensity and luminance of R increased by two‐ and one‐fold, respectively. Additionally, the afterglow duration extended from 9 to 17 min. This hybrid strategy presents novel ideas and methods to enhance the afterglow performance of persistent‐red inorganic phosphors. Due to its excellent afterglow performance and simple preparation, R@TCPP 100:1, when combined with HDPE and processed into thin‐film rolls, can be utilized in sound‐controlled afterglow lamps. This makes it highly appealing for applications such as flexible large‐area luminescence, wearable devices, and low‐vision lighting.

## 4 Experimental Section

### Preparation of R@TCPP Solid Powder

The preparation method for R@TCPP was outlined as follows: TCPP (0.100, 0.020, 0.010, 0.005, or 0.003 g) was placed in a beaker, and 60 mL DMF was added. The mixture was sonicated for 5 min to ensure complete dissolution of TCPP in DMF. Subsequently, Sr_0.75_Ca_0.25_S: Eu^2+^ (1 g) was added, heated, and stirred (temperature: 90 °C, speed: 650 rad min^−1^) for 9 h. The product was then filtered and placed in an oven at 80 °C for 12 h, and dried thoroughly to obtain solid powder R@TCPP (10:1, 50:1, 100:1, 200:1, and 300:1).

### Preparation of R with Different Particle Sizes

Sr_0.75_Ca_0.25_S: Eu^2+^ (R) was uniformly dispersed in DMF and filtered through three molecular sieves with varying pore sizes (repeated 3 times). The samples were subsequently oven‐dried at 80°C for 12 h.

### Chemical Structure Characterization

Powder X‐ray diffraction (XRD) measurements were recorded on a SmartLab SE using Cu Kα radiation with 2θ range of 10°–90°, 40 KeV, and 50 mA at a scanning rate of 10 min^−1^ (2θ) at room temperature. X‐ray photoelectron spectroscopy (XPS) was performed on a Thermo ESCALAB 250XI. Fourier‐Transform Infrared Spectrometer (FT‐IR) spectra were recorded using a Nicolet Is‐10 FT‐IR Spectrometer. Scanning electron microscopy (SEM) images were obtained with a FEI Quanta 4000 FEG.

### Photophysical Characterization

Phosphorescence spectra, fluorescence spectra, and excitation spectra were measured at both room temperature and 77 K on a Hitachi F‐4700 fluorescence spectrophotometer. Afterglow spectra and afterglow intensity decay curves at room temperature were measured on an F97pro fluorescence spectrophotometer. This test was carried out after closing the light gate and manually irradiating with a UV lamp and removing the lamp (afterglow spectra: excitation wavelength: 365 nm, power: 30 W, excitation time: 5 s, fast scanning for five times in 10 s; afterglow intensity decay curves: excitation wavelength: 365 nm, power: 30 W, excitation time: 30 s, scanning time: 10 min). The afterglow luminance decay curve at room temperature was measured by a PR‐305 detector (excitation time: 3 min; steady state interval: 0.1 s; scan time: 10 min). DLS testing was performed on a Mastersizer 2000. UV–vis absorption spectra were recorded on a Lambda 650 UV–vis–NIR spectrophotometer. Thermoluminescent spectra were measured on an FJ‐427A1 Microcomputer thermoluminescent dosimeter (excitation wavelength: 365 nm, power: 30 W, excitation time: 3 min, temperature range: 30 −300 ^o^C, temperature rise rate: 1 °C s^−1^). The HDPE@R@TCPP 100:1 film was fabricated using an RM‐200A torque rheometer. The sound‐controlled afterglow light was powered by two 1.5 V dry cell No.5 batteries, and the LED operating voltage was 2 V. Photographs and videos were captured with a Canon EOS 80D camera.

## Conflict of Interest

The authors declare no conflict of interest.

## Author Contributions

Y.K.W. and C.L.Y. conceived and were responsible for the experiments. Y.K.W. and Q.K.L., synthesized the materials and performed the photoluminescence measurements. Y.Z., J.Y.H., C.L., Y.Z., and Q.A.C. completed the preparation of the application. L.J.Q. performed theoretical calculations. Y.K.W. and C.L.Y. performed the data analysis and wrote the manuscript. All authors contributed to the final version of the manuscript.

## Supporting information

Supporting Information

Supplemental Movie 1

Supplemental Movie 2

Supplemental Movie 3

Supplemental Movie 4

## Data Availability

The data that support the findings of this study are available in the supplementary material of this article.
