# Supplementary material for: Effective Long Afterglow Amplification Induced by Surface Coordination Interaction
Source: Adv Sci (Weinh). 2023 Dec 31;11(11):2306942. doi: 10.1002/advs.202306942 (PMC10953560; doi:10.1002/advs.202306942)
Supplement: Supplementary file 1 — Supporting Information [file ADVS-11-2306942-s001.pdf]

ADVANCED SCIENCE
Open Access

Supporting Information

for *Adv. Sci.*, DOI 10.1002/advs.202306942

Effective Long Afterglow Amplification Induced by Surface Coordination Interaction

*Yongkang Wang, Qiankun Li, Lunjun Qu, Jiayue Huang, Ying Zhu, Chen Li, Qingao Chen, Yan Zheng and Chaolong Yang**

Supporting Information

Effective long afterglow amplification induced by surface coordination interaction

*Yongkang Wang, Qiankun Li, Lunjun Qu, Jiayue Huang, Ying Zhu, Chen Li, Qingao Chen, Yan Zheng, Chaolong Yang**

Table of Counts

EXPERIMENTAL PROCEDURES 3

I. REAGENTS AND MATERIALS 3

II. THEORETICAL CALCULATION METHOD 4

III. SUPPLEMENTARY FIGURES AND TABLES 5

IV. SUPPORTING MOVIES 17

Experimental Procedures

I. Reagents and materials

Unless otherwise stated, following regents used in experiments including 1,3,6,8-Tetra(4-carboxylphenyl) yrene (TCPP), 2,6-Naphthalenedicarboxylic Acid (26NC) and 5,5',5'',5'''-(Pyrene-1,3,6,8tetrayl)tetraisophthalic acid (PTTA) were purchased from Energy Chemical without further purification. High density polyethylene (HDPE) was purchased from Suzhou Qiangyoufa Plastic Chemical. N,N-Dimethylformamide (DMF, Analytical Reagent) was purchased from KeLong chemical. $Sr_{0.75}Ca_{0.25}S$: Eu^{2+} (R) was purchased from LuMing Technology Group Limited.

Structural formula of organic ligands

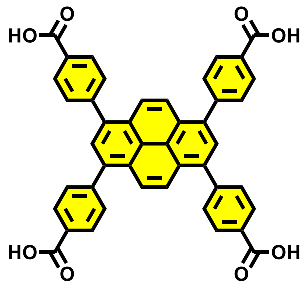

1,3,6,8-Tetra(4-carboxylphenyl) pyrene (TCPP)

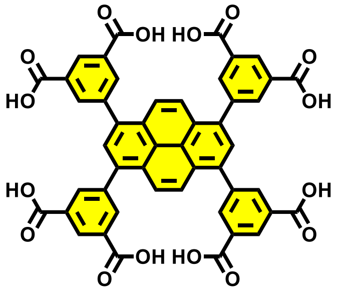

5,5',5'',5'''-(Pyrene-1,3,6,8tetrayl) tetraisophthalic acid (PTTA)

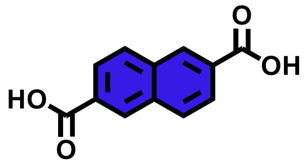

2,6-Naphthalenedicarboxylic Acid (26NC)

II. Theoretical calculation method

For the theoretical simulations, the geometry of the ground state in the isolated state was fully optimized with density functional theory (DFT) with B3LYP hybrid functional at the basis set level of 6-31G**. All the excited state geometries were optimized by the time-dependent DFT (TD-DFT) with B3LYP functional at the same basis set level as the ground state. The excitation energy in the n-th singlet (Sn) and n-th triplet (Tn) states of monomer was obtained by TD-DFT calculations on the M062X/6-31G(d,p) level of theory. All the above calculations were performed using ORCA 5.0.4 program package.

III. Supplementary figures and tables

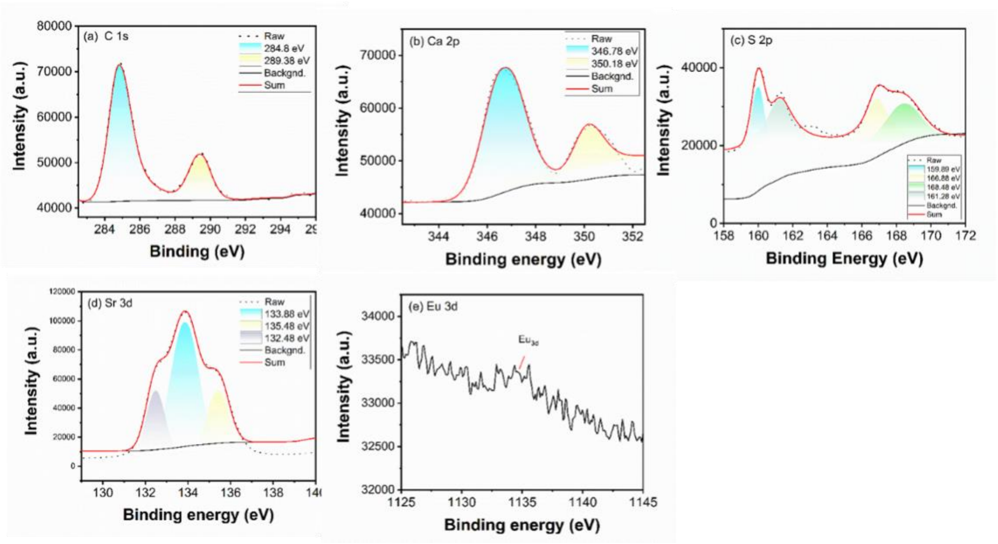

Figure S1. XPS high-resolution C_{1s}, Ca_{2p}, S_{2p}, Sr_{3d} and Eu_{3d} spectra of R.

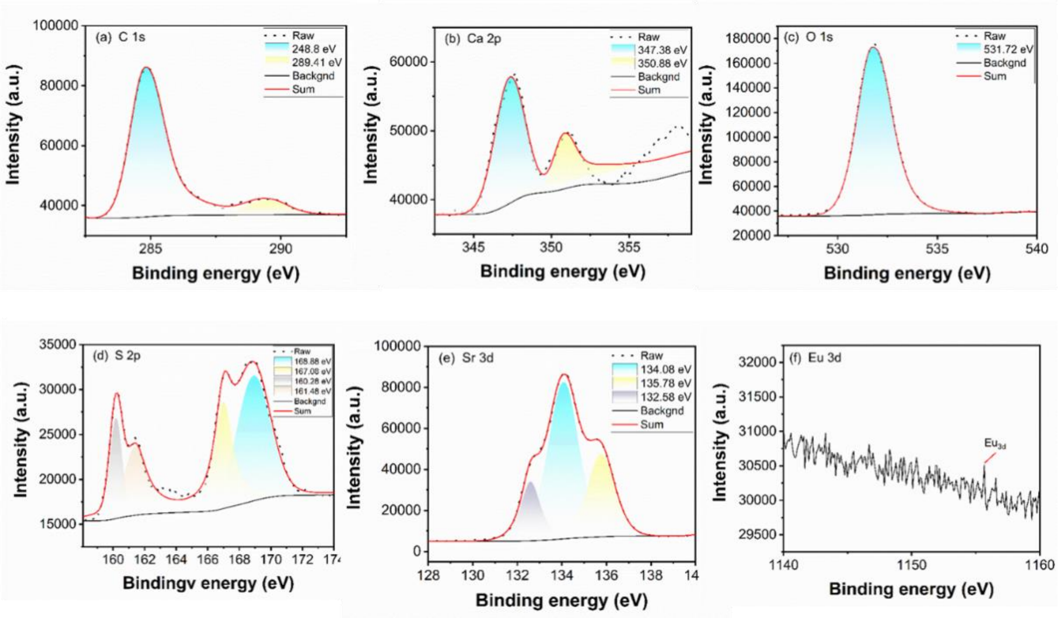

Figure S2. XPS high-resolution C1s, Ca2p, O1s, S2p, Sr3d and Eu3d spectra of R@TCPP 100:1.

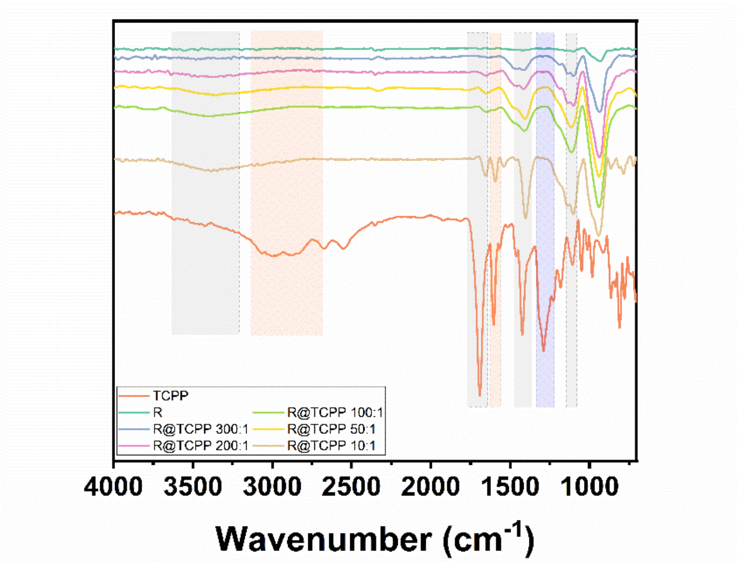

Figure S3. FT-TR spectral studies. FT-TR spectra of R and R@TCPP series ratios.

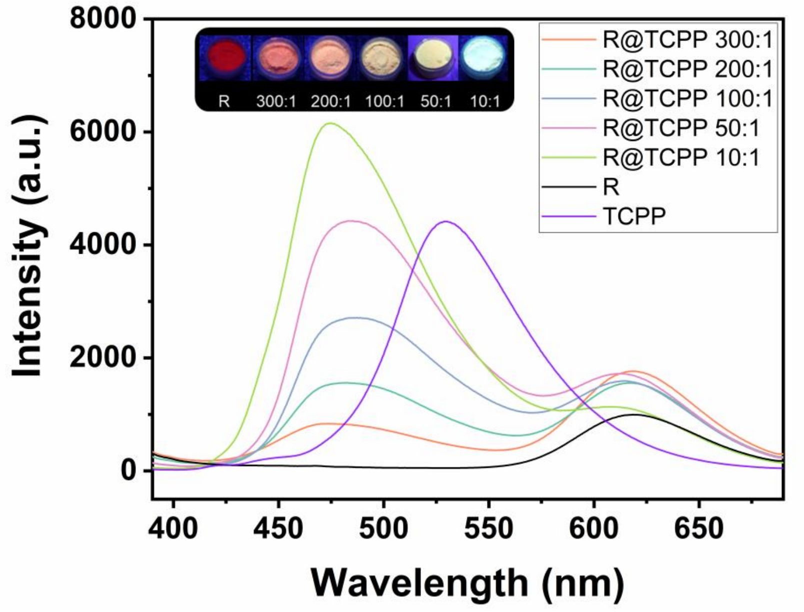

Figure S4. Fluorescence spectra of R and R@TCPP series ratios.

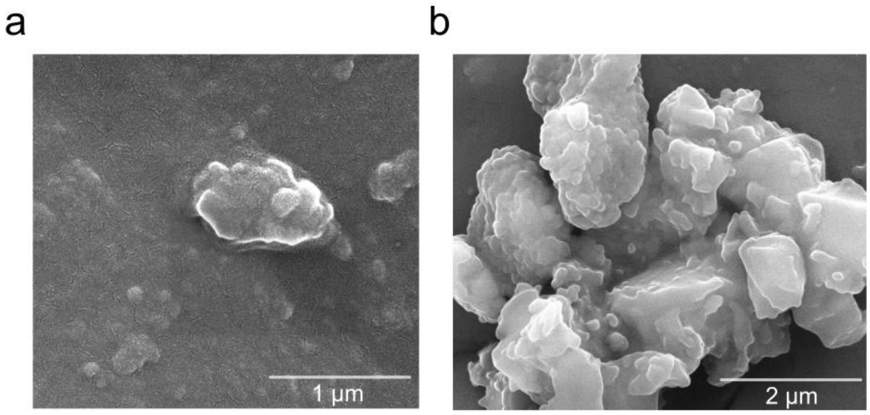

Figure S5. Characterization of the R and R@TCPP 100:1. a, SEM image of the R. b, SEM image of the R@TCPP 100:1.

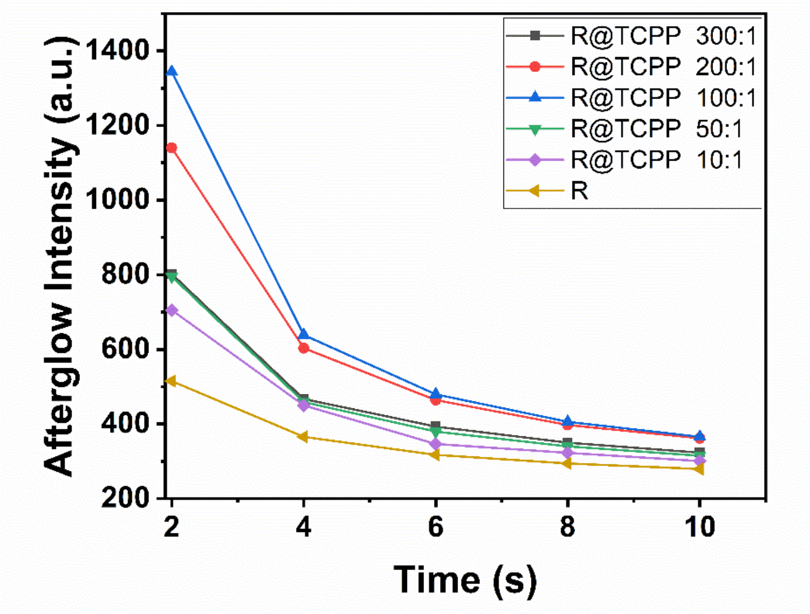

Figure S6. Decay curves of afterglow intensity (λem. = 625 nm) of R and R@TCPP series samples. The curves are obtained from five scans in 10 s after removing the UV lamp.

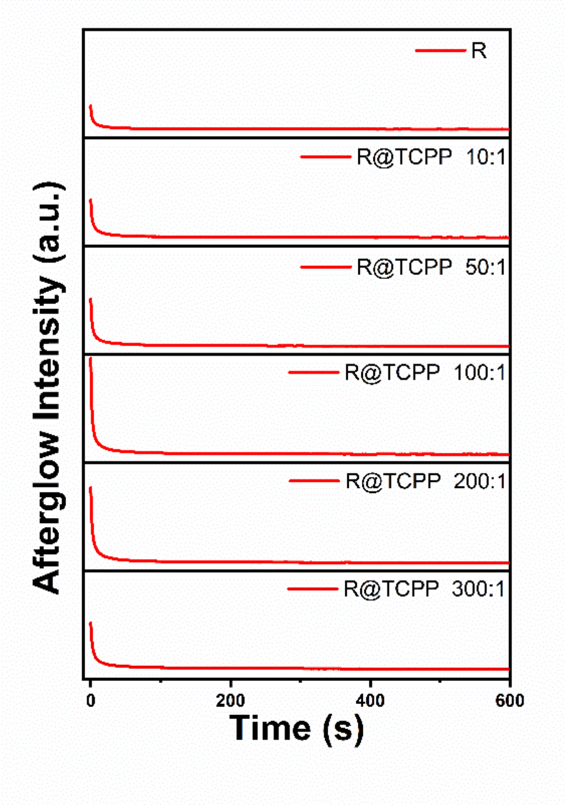

Figure S7. Afterglow intensity decay curves of R and R@TCPP series ratio.

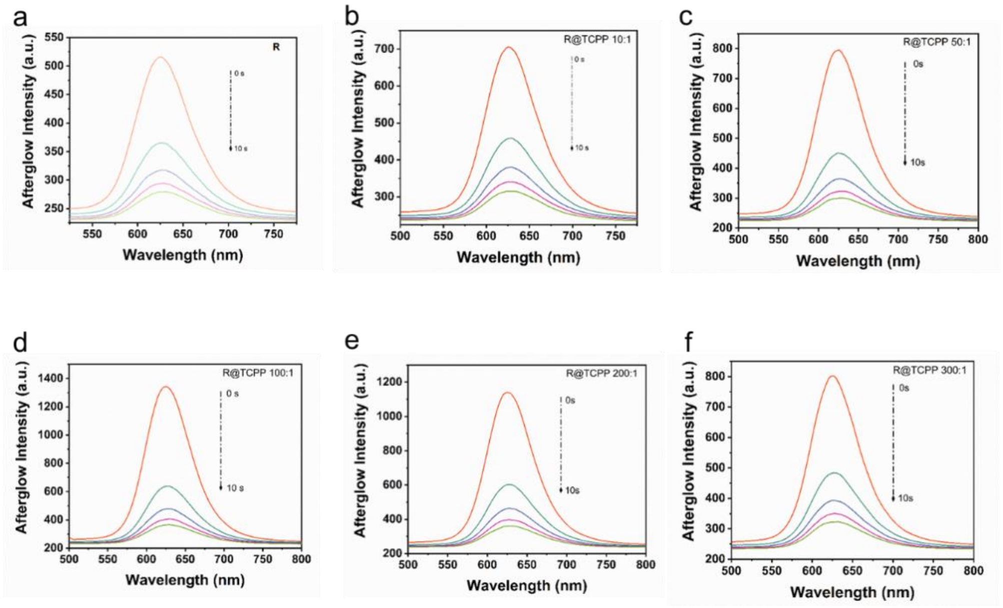

Figure S8. Afterglow spectra were obtained by scanning the sample five times within 10 s after removing the UV lamp. The afterglow spectrum of (a)R, (b)R@TCPP 10:1, (c) R@TCPP 50:1, (d) R@TCPP 100:1, (e) R@TCPP 200:1, (f) R@TCPP 300:1**.**

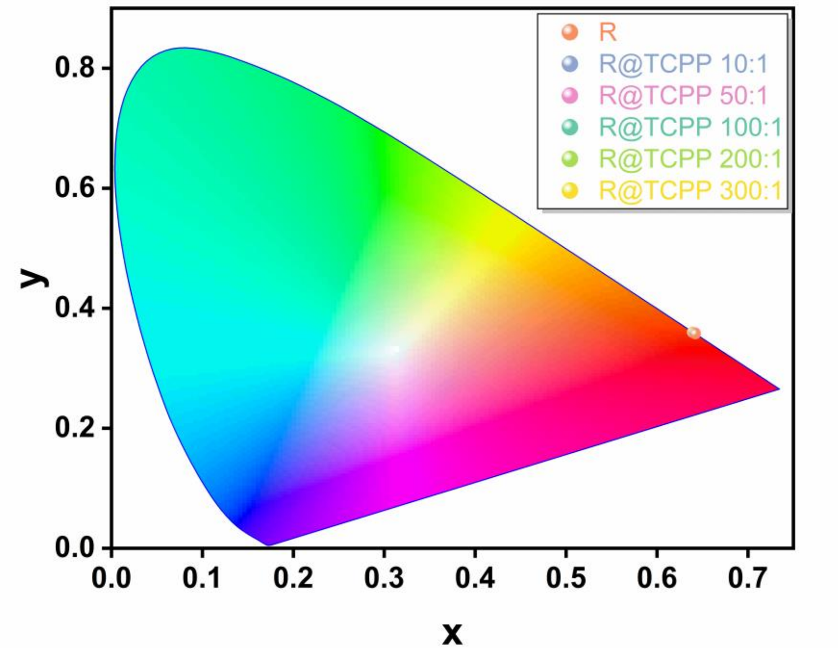

Figure S9. CIE coordinate diagrams of R, R@TCPP 10:1, R@TCPP 50:1, R@TCPP 100:1, R@TCPP 200:1, R@TCPP 300:1.

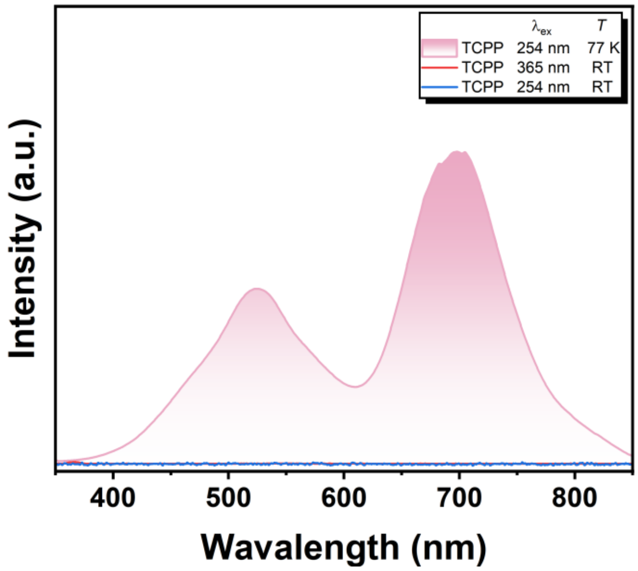

Figure S10. Phosphorescence spectrum of TCPP at 77 K and room temperature.

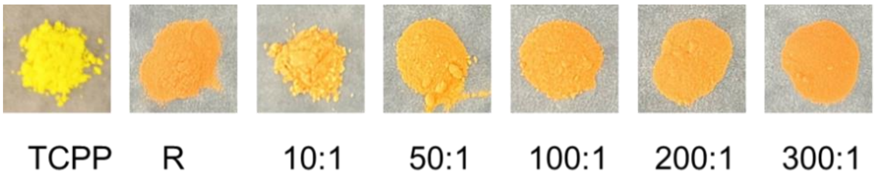

Figure S11. Daylight photos of TCPP, R and R@TCPP series ratio.

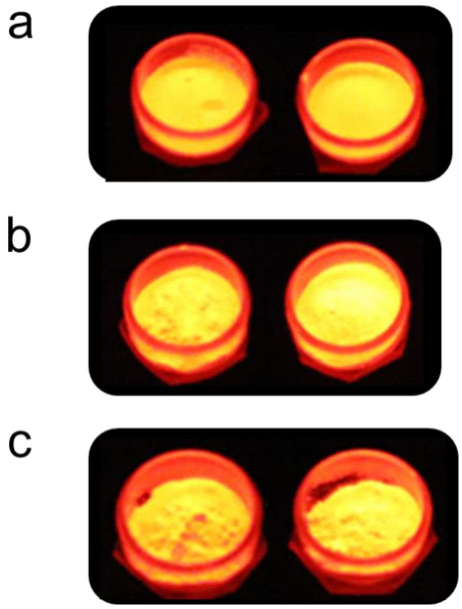

Figure S12. Afterglow photos before (left) and after (right) R modification for different particle sizes. a, the particle sizes is 44 μm. b, the particle sizes is 25 μm. c, the particle sizes is 12 μm.

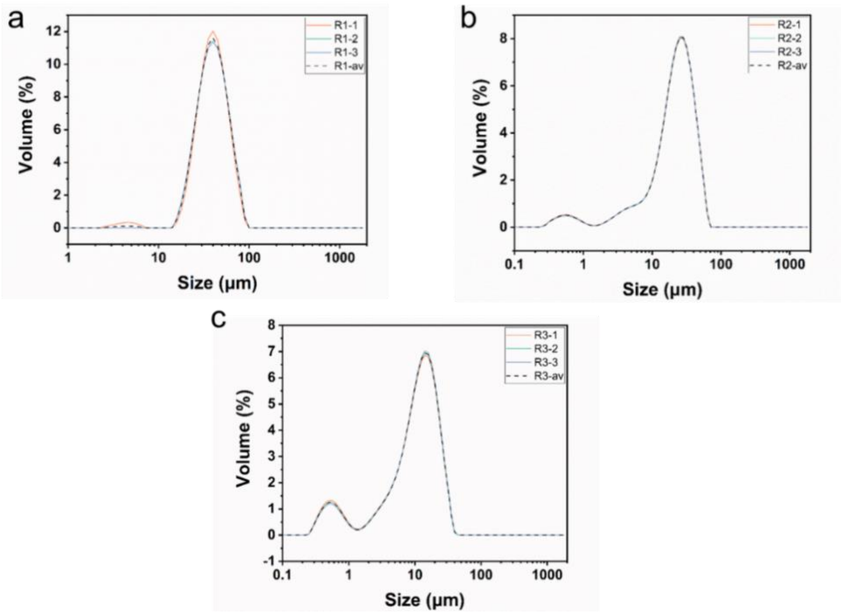

Figure S13. DLS curves of R solid powders with different average particle sizes under DMF dispersions, the four curves are the three scanned values and the average value. a, R1, the average particle size is 44 μm. b, R2, the average particle size is 25 μm. c, R3, the average particle size is 12 μm.

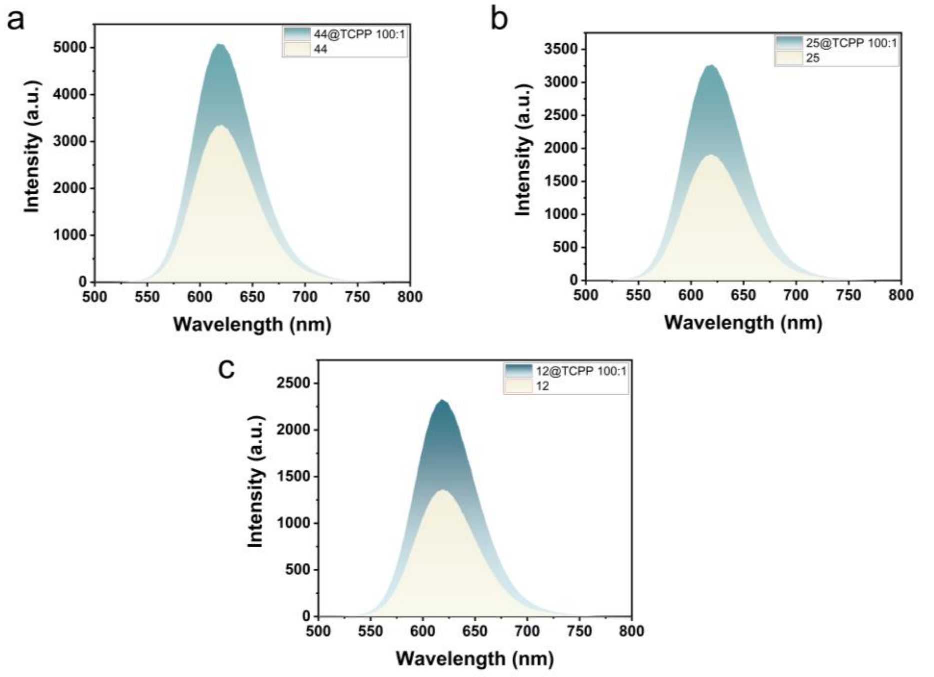

Figure S14. Phosphorescence spectra of R and R@TCPP 100:1 for different average particle sizes. a, Average particle size 44 μm. b, Average particle size 25 μm. c, Average particle size 12 μm.

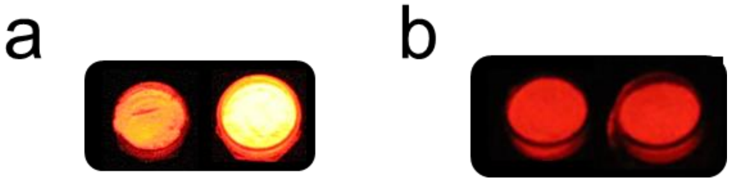

Figure S15. Comparison afterglow photos of R and R@TCPP 100:1 after excitation by different light sources. a, 365 nm UV lamp, Power is 30 w, Excitation time is 2 s. b, LED lights for smartphones, Excitation time is 2 s.

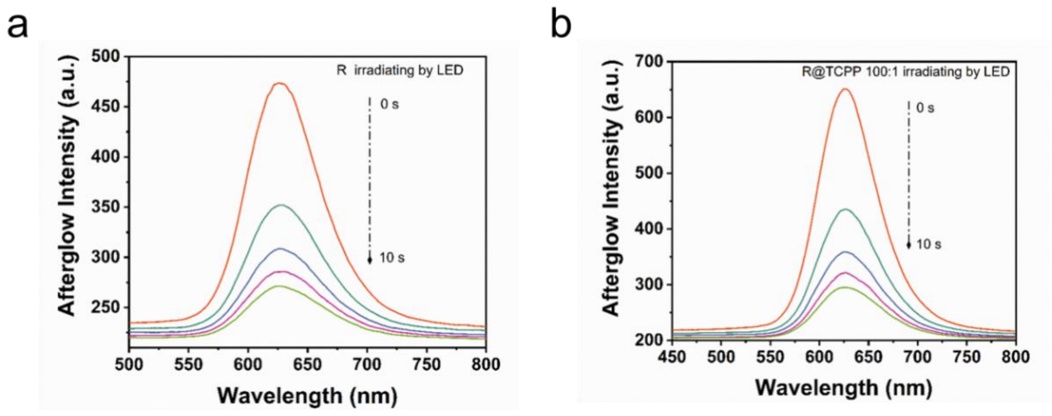

Figure S16. Afterglow spectra were obtained by scanning the sample five times within 10 s after removing the UV lamp, the figure shows the spectrum obtained from the first scanning, the excitation lamp source is LED light of smart phones, irradiation time is 5 s. The afterglow spectrum of (a) R, (b) R@TCPP 100:1.

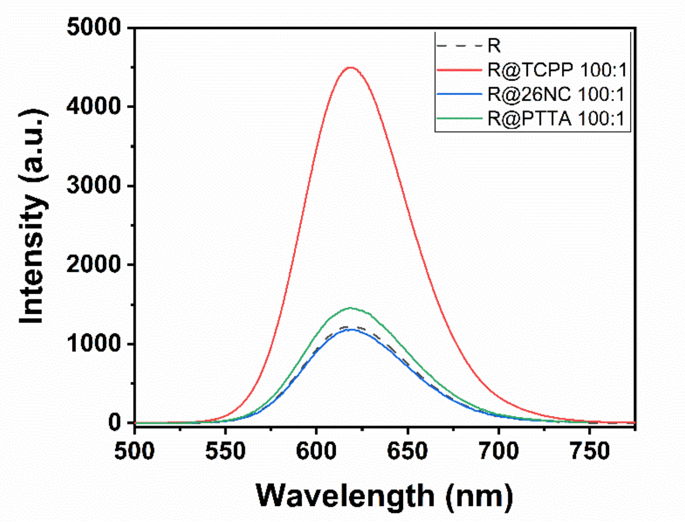

Figure S17. Phosphorescence spectrum of R, R@TCPP 100:1, R@PTTA 100:1 and R@26NC100:1.

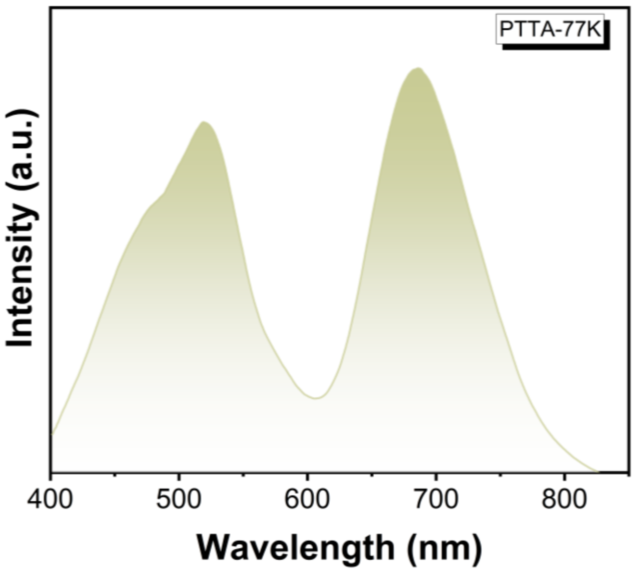

Figure S18. Phosphorescence spectrum of PTTA at 77 K(λex.= 254 nm)

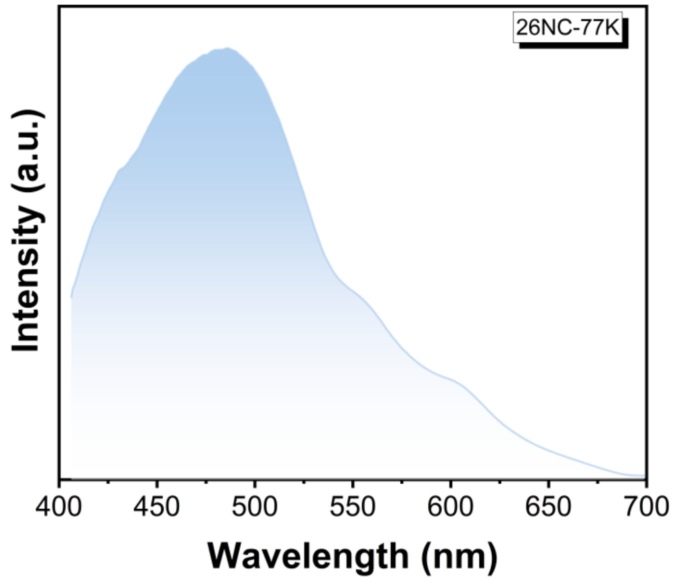

Figure S19. Phosphorescence spectrum of 26NC at 77 K(λex.= 365 nm).

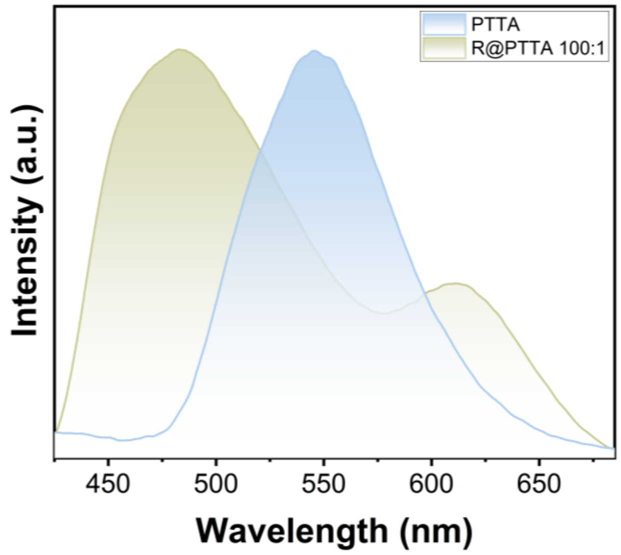

Figure S20. Normalized fluorescence spectra of TPPA and R@TPPA 100:1 at room temperature (λex.= 365 nm).

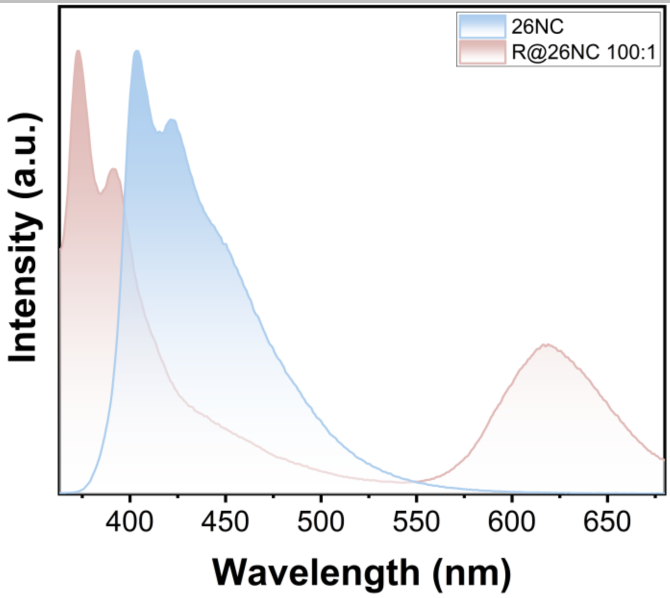

Figure S21. Normalized fluorescence spectra of 26NC and R@26NC 100:1 at room temperature (λex.= 350 nm).

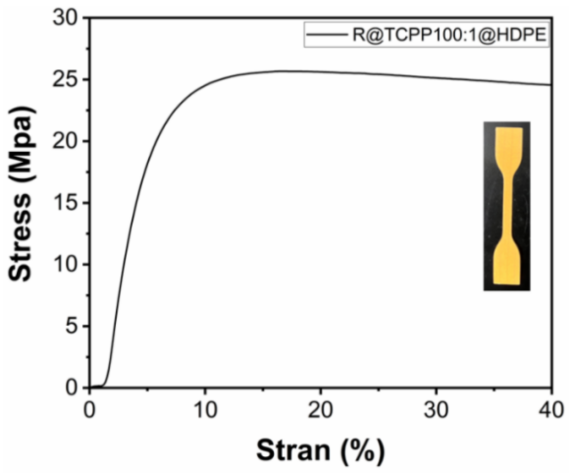

Figure S22. The stress-strain curve of R@TCPP100:1@HDPE (The photo in the figure is the test spline. The thickness of the spline is 0.29 mm, and the width of the test area is 4 mm).

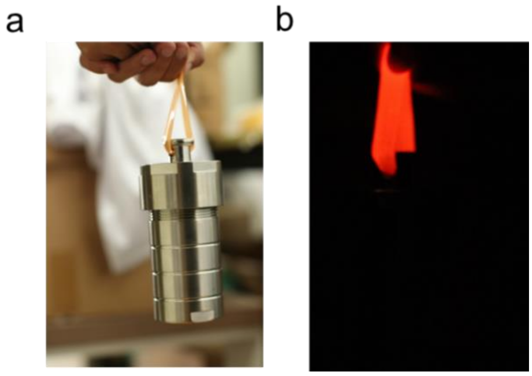

Figure S23. The real photo and afterglow photo of HDPE@R@TCPP 100:1 film when pulling the reactor, □ex.=365 nm, Power =30W, the irradiation time is 2 s.

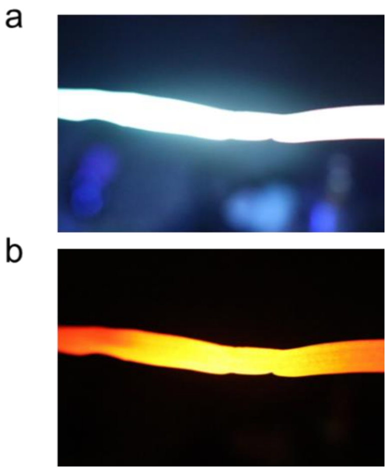

Figure S24. Fluorescence photo and afterglow photo of HDPE@R@TCPP 100:1 after pulling the reactor, □$\lambda_{ex.}$=365 nm, Power =30 W, the irradiation time is 2 s

Tables S1. Percentage content of each element in R and R@TCPP 100:1

**R**		**R@TCPP 100-1**	
Element	At%	Element	At%
C	9.36	C	14.32
S	27.28	S	6.39
Sr	4.36	Sr	4.24
Ca	2.32	Ca	2.06
O	54.15	O	72.99
Eu	0.02	Eu	0.03

IV. Supporting movies

Movie S1. Afterglow movie of R and R@TCPP series samples. The samples were all solid powders, from left to right are R@TCPP 300:1, R@TCPP 200:1, R@TCPP 100:1, R@TCPP 50:1, R@TCPP 10:1and R, the excitation wavelength is 365 nm, the power of UV lamp is 5 W, irradiation time is 10 s. The movie is played at five times the speed, the afterglow time of R@TCPP 100:1 and R are 17 min and 9 min, respectively.

Movie S2. Afterglow movie of R, R@TCPP 100:1, R@TPPA 100:1 and R@26NC 100:1. The samples were all solid powders, the excitation wavelength is 365 nm, the power of UV lamp is 5 W, irradiation time is 10 s. The movie is played at five times the speed, the afterglow time of R, R@TCPP 100:1, R@TPPA 100:1 and R@26NC 100:1. are 9 min, 17 min, 12 min and 9 min, respectively.

Movie S3. Afterglow movie of R@TCPP 100:1@HDPE film. The excitation wavelength is 365 nm, the power of UV lamp is 30 W, irradiation time is 5 s. The movie is played at five times the speed, the afterglow time of R@TCPP 100:1@HDPE film is 8 min.

Movie S4. Afterglow movie of sound-controlled afterglow lamp. The movie is played at two times the speed, the afterglow time of sound-controlled afterglow lamp is 6 min.n
